# Development of the Parent-to-Infant Bonding Scale: Validation in Swedish Mothers and Fathers in Community and Clinical Contexts

**DOI:** 10.1007/s10578-024-01699-x

**Published:** 2024-05-17

**Authors:** Sara Lindeberg, Eva Tedgård, Birgitta Kerstis, Ulf Tedgård, Alyx Taylor, Peter Jönsson

**Affiliations:** 1 Department of Health Sciences, Child and Family Health, Faculty of Medicine, Lund University, Lund, Sweden; 2 Scania Regional Council, Department for Regional Development, Malmö, Sweden; 3 Department of Clinical Sciences Lund, Child and Adolescent Psychiatry, Faculty of Medicine, Lund University, Lund, Sweden; 4 Skåne University Hospital, Child and Adolescent Psychiatry, Infant and Toddler Unit, Malmö, Sweden; 5 Division of Caring Sciences, School of Health, Care and Social Welfare, Mälardalen University, Västerås, Sweden; 6 Department of Clinical Sciences Lund, Paediatrics, Faculty of Medicine, Lund University, Lund, Sweden; 7 Department of Paediatrics, Skåne University Hospital, Lund, Sweden; 8 School of Health and Rehabilitation Sciences, AECC University College, Bournemouth, UK; 9 Department of Psychology, Faculty of Education, Kristianstad University, Kristianstad, Sweden

**Keywords:** Father-infant bonding, Mother-infant bonding, Mother-to-Infant Bonding Scale (MIBS), Parent-to-Infant Bonding Scale (PIBS), Postpartum Bonding Questionnaire (PBQ)

## Abstract

Valid measurement instruments are needed to investigate the impact of parental bonding on child health development. The aim was to develop and validate a self-report questionnaire, the Parent-to-Infant Bonding Scale (PIBS) to measure bonding in both mothers and fathers. Internal consistency and construct validity were analysed using data from Swedish parents from both clinical (*N* = 182), and community (*N* = 122) population samples. Overall, good or acceptable internal consistency of the PIBS appeared. Convergent validity (against the Postpartum Bonding Questionnaire, analysed in the clinical sample) and discriminant validity (against the mental health constructs of depressive symptoms and anxiety) were demonstrated. The results support the PIBS as a measure of maternal and paternal bonding in community and clinical populations. Assessments of criterion validity in these populations are desirable. The similarities in PIBS measurement properties between the parent groups suggest its usefulness for comparisons between mothers and fathers, and for future investigations of unique and interactive impacts of maternal and paternal bonding on child outcomes using community and clinical cohorts.

**Supplementary Information:**

The online version contains supplementary material available at 10.1007/s10578-024-01699-x.

## Introduction

It is widely recognised that the early developmental period, from conception and through the child’s first years, is crucial for setting the foundation of a child’s future well-being [1, 2]. It is also generally agreed upon that children’s early development requires nurturing care, including responsive caregiving to prevent an accumulation of adversities disrupting brain development, infant attachment, and early learning, a process which can continue throughout the life-course [3]. Maternal mental health is an established factor influencing child development [4]. Another example is a mother’s ability to have positive representations of or bond with her child with implications for the child’s health and wellbeing during infancy, for example attachment quality [5, 6]. However, Le Bas et al. [6] identified in their systematic review a significant knowledge gap and a paucity of studies investigating the link between bonding and infant outcomes. Furthermore, there is evidence suggesting that maternal bonding disorders are more common for older children, studied in different age groups up to eight years possibly indicating that early difficulties in the parent-infant relationship may worsen over time [7], but this idea has not been investigated in prospective longitudinal studies. To investigate these processes further in both community and clinical contexts, as well as the link between early bonding and more long-term child outcomes during child- and adulthood from a life-course perspective, and also to evaluate relevant interventions in these contexts, there is a need for valid self-report measures of parental bonding [7, 8].

The concept of bonding has mainly been used in the context of motherhood and the definition remains unclear [9, 10]. In addition, formal diagnostic criteria for bonding disorders are yet to be determined [7, 11]. Several scientific concept analyses of maternal bonding have defined mother-infant bonding as a process in which the maternal feelings and emotions towards the infant are its primary indicators, often described as occurring in the first week or year of an infant’s life [12, 13]. However, the bonding process is also conceptualised as starting during pregnancy [14] and bonding is frequently measured prepartum [8, 15]. Therefore, parent-infant bonding is currently used broadly to describe the parent’s internal experiences regarding their child, whether born or unborn [9].

Bonding is semantically often used interchangeably with attachment in the bonding literature [16], but as a scientific concept the latter was initially described as encompassing the infant’s tie with and behaviours towards the caregiver [17, 18]. Several authors therefore define bonding and attachment as representing different phenomena [13, 16, 19, 20], with bonding representing the caregiver’s tie with the child, and attachment representing the child’s tie with the caregiver. Bonding is also defined as separate from constructs of mental health such as depression and anxiety [13, 21].

Qualitative research has demonstrated similar bonding processes for fathers and for mothers although differences between them have appeared, for example in terms of being a slower process for fathers [22, 23]. While the biological and behavioural components unique to mother-infant bonding remain to be fully explored [9, 13], research suggests that the components of the maternal bonding process differ from the corresponding components of paternal bonding as described in a review by Abraham and Feldman [24]. It was argued that maternal and paternal bonding nevertheless are equally important and that the respective behavioural parenting characteristics associated with maternal and paternal bonding complement each other in their contributions to infant attachment and cognitive, social, and emotional growth [24]. The dominant conceptualisation of bonding as a parental emotional process driven by the feelings towards the child is mainly operationalised through study questionnaires [7, 8, 13]. Previous research covering the prepartum period and the first two postpartum years has demonstrated similarities in its characteristics between mothers and fathers [25–30]. Bonding conceptualised in terms of its emotional dimension thus appears to encompass a common denominator of maternal and paternal bonding, independent from its respective biological underpinnings and behavioural manifestations, making it appropriate for further use within the contexts of both motherhood and fatherhood.

A systematic review of parental pre- and postpartum bonding measures revealed that psychometrically robust bonding instruments for research and clinical practice are yet to be developed or determined [8]. The aim of this study was to validate a modified version of the Mother-to-Infant Bonding Scale [20] in Swedish mothers and fathers, referred to as the Parent-to-Infant Bonding Scale assessing parents’ feelings towards their child. The measurement properties assessed were internal consistency, and construct validity including hypotheses testing [31], using the Postpartum Bonding Questionnaire [32] and measures of depressive symptoms and anxiety as comparator instruments.

## Methods

### Participants and Study Designs

The study involved two data collections with different designs. One data collection was a longitudinal infant-parent cohort study with parents recruited during pregnancy from a community population. The other one was a cross-sectional study with data collected from parents about to receive treatment at an infant mental health unit for dysfunctions concerning the relationship with their child.

#### Community Population Sample of Parents from the Scania Birth Cohort

The parents in the community sample were recruited during the third trimester of pregnancy for participation in the longitudinal “Scania Birth Cohort (SBC) pilot study—Health development in a life-course perspective” aiming to validate measurements of early-life factors and predictors of health development during the child’s first three years, including parental factors, and investigate associations between these factors. Exclusion criteria were multiple fetus pregnancy, donor egg pregnancy, insufficient skills in the Swedish language, and premature births. The SBC pilot study includes five waves of data collection, the first one during pregnancy and the last one when the child has turned three years old. The participants of the present study were 65 mothers and 57 fathers from the second to fourth waves of the SBC study when the children were between 1 and 12 months old and enrolled in standard national postnatal child health services (CHS) care, free of charge. Data were collected at a child health centre in Scania in southern Sweden, at the regular CHS visits taking place at infant ages 4–6 weeks, 6 months, and 12 months, here referred to as Time 1, 2, and 3 (T1–T3) respectively. This data collection took place between May 2019 and June 2021. Two families terminated their participation in the study during follow-up rendering a drop-out rate of 2.6%. Due to unforeseen obstacles including the prolonged restrictions connected with the COVID-19 pandemic, all data collection taking place at the child health centre was terminated in June 2021.

#### Clinical Population Sample of Parents from an Infant Mental Health Unit

The study participants in the clinical sample were recruited from parents who were about to receive specialist psychotherapeutic care at an infant mental health (IMH) unit treating families with children in the age range of 0–4 years, free of charge. The aims of the IMH unit involve improving the parent–child relationship and identifying potential psychiatric symptoms influencing the parents’ caregiving abilities at an early stage (with potential pharmacological treatment provided outside of the IMH). The psychotherapeutic treatment at the IMH is designed to support the parents in developing greater sensitivity to the child’s needs and increasing the ability to reflect on the child’s expressed affect and intentions [33]. The study participants were recruited to validate a variety of parental self-report measurements of mental health and bonding that were being introduced as part of the unit’s clinical tools. Exclusion criteria were psychosis or severe depression with hospitalisation, and insufficient Swedish language skills. The data collection was performed between April 2019 and June 2021. The surveys were sent home by post before the first treatment session, after a specialist care need had been identified, to each parent in the family irrespectively of clinical assessments of individual treatment needs. Three parents were foster parents, and since there was no information on how long they had the child in their care they were excluded from analysis yielding 100 mothers and 82 fathers for the present study.

### Assessment Measures

#### Development of the Parent-to-Infant Bonding Scale

The Parent-to-Infant Bonding Scale (PIBS) was developed from the Mother-to-Infant Bonding Scale (MIBS) [20], an eight-item self-report scale consisting of one-word items developed to measure bonding in biological mothers. The MIBS was devised as a simple measure of the unidirectional feelings of the mother towards her infant without requiring response or activity by the infant, making it available for use from day one postpartum and even prepartum [34]. It was intended to be used in the general population to capture bonding problems for research and screening purposes [20]. The terms in the questionnaire were derived from research using qualitative methodology with women who had sought medical help for difficulties with the early relationship with their infants [35].

**Fig. 1:**
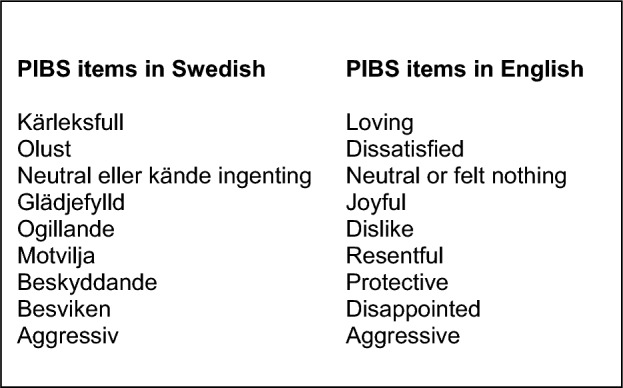
The Parent-to-Infant Bonding Scale (PIBS) items in Swedish and English

For the present project, the MIBS items appeared to exhibit face validity for measuring bonding in other parent groups besides biological mothers, including fathers. First, the MIBS was translated to Swedish by the first author (SL), and back-translated to English by a native English bilingual midwife researcher not previously familiar with the questionnaire. It was found that for one word, “Resentful”, it was challenging to find a Swedish translation fitting the context. Furthermore, several previous studies on the MIBS [36–38] found rather low internal reliability coefficients of the MIBS, and since the magnitude of the internal reliability coefficient value partly relies on the number of scale items [39], it was considered that inclusion of an additional item may alleviate this issue. Therefore, following discussions between the first and second authors (SL and ET), two Swedish items, *Olust* and *Motvilja*, were introduced to represent “Resentful”. The new nine-item scale is referred to as the Parent-to-Infant Bonding Scale or PIBS, and the Swedish item *Olust* is here translated to “Dissatisfied” for the English version of the PIBS (Fig. 1). The respondents rate the degree of agreement to the PIBS statements on a 4-point Likert scale [Supplementary Information (SI 1)], with the positive items “Loving”, “Joyful”, and “Protective” scored as 0–3 with 0 representing “I agree very much” and 3 representing “I disagree totally”, and the six negative items including for example “Neutral or felt nothing” and “Aggressive” reverse-scored. The final score is the sum of all nine items with a total possible score ranging between 0 and 27, the higher the score the higher the risk of bonding difficulties.

In the community sample at T1, the parents were asked in the questionnaire to recall their feelings towards their child in the “first weeks” after childbirth, here referred to as PIBS first weeks, whereas at T2 and T3 they were asked about how they felt towards their child “now”, referred to as PIBS current [Supplementary Information (SI 1)]. For the clinical sample the PIBS was included twice in the survey; the parents were first asked to recall their feelings towards their child in the first weeks after the child was born (PIBS first weeks), and then they were asked about their feelings towards their child now (PIBS current).

#### The Postpartum Bonding Questionnaire

The Postpartum Bonding Questionnaire (PBQ) was developed to screen for maternal bonding disorders in obstetric and primary care [32]. It consists of 25 items on which the respondents rate their agreements of statements on a 6-point Likert scale, the higher the score the higher the risk of bonding difficulties. The PBQ includes four subscales labelled impaired bonding (12 items), rejection and anger (7 items), anxiety about care (4 items), and risk of abuse (2 items) [32]. In contrast to the PIBS, the PBQ contains items presupposing response by the infant, e.g., “I feel happy when my baby smiles or laughs” making it unsuitable for use during pregnancy and in the first few postpartum weeks before such response is developmentally possible.

The PBQ was intended to be included in the questionnaires for both population samples, but the ethics committee prevented its inclusion in the community sample. This was due to that the committee found it unethical to gain information that parents may be at risk of harming their child while not having the means to act on it. The committee referred to specific PBQ items, for example the impaired bonding subscale item “I wish my baby would somehow go away”, as well as the risk of abuse items. The two-item PBQ risk of abuse subscale has demonstrated poor psychometric properties, and it has been suggested to omit this from the PBQ [38]. Therefore, a 23-item version of the PBQ including three of the four original subscales [Supplementary Information (SI 2)] was used in the clinical sample as a current measure of bonding. For the statistical analyses PBQ total and subscale scores were summed up.

Two Swedish translations of the PBQ [26, 29], not formally validated [8] have been used for scientific research. Ten items from one of these translations [29] were modified by SL and ET to optimise the translation and comprehensiveness of the current Swedish version of the PBQ, the other 13 items were used without modification. The item “I feel trapped as a mother” was changed to “I feel trapped as a parent” adjusting the instrument for usage in any parental population. A native Canadian living in Sweden conducted a back-translation [Supplementary Information (SI 2)], which was sent to and acknowledged by Professor Ian F Brockington who developed the PBQ.

#### Measurements of Depressive Symptoms and Anxiety

The Swedish [40] 21-item version of the Depression Anxiety Stress Scales (DASS-21), designed to measure the emotional states of depression, anxiety, and stress in adults with three seven-item self-report scales [41], was included in the surveys for both population samples. The DASS depression (DASS-D) and anxiety (DASS-A) scales, scored on a 4-point Likert scale with higher scores indicating higher levels of depressive or anxiety symptoms were used in the current analyses. In the surveys for the community sample, the Edinburgh Postnatal Depression Scale (EPDS) [42, 43] was also included. The EPDS is a ten-item self-report questionnaire scored on a 4-point Likert scale with higher scores indicating higher levels of depressive symptoms. It has been validated to screen for pre- and postpartum depression in both mothers [44] and fathers [45].

### Statistical Analysis

Analyses were guided by the Consensus-based standards for the selection of health status measurement instruments (COSMIN), an initiative to improve and standardise the development and selection of health measurements [31]. According to COSMIN, aspects of an instrument’s validity are referred to as “measurement properties” [31, 46–48]. The measurement properties internal consistency and construct validity were assessed, including hypotheses testing as part of the assessments of construct validity [31, 49].

The PIBS bonding data were examined using tests for normality and histograms revealing non-normally distributed data. Therefore, Mann–Whitney U was used for testing differences in bonding scores between groups, and robust one-way repeated measures ANOVA with planned contrasts (T1 vs. T2; T2 vs. T3), with Bonferroni correction for multiple tests was used for the change in community groups over time. Wilcoxon signed-rank test was used for testing differences between timepoints within clinical groups. Spearman’s rank correlation test was used to determine cross-correlations.

Internal reliability coefficients were calculated for all measurements including the comparator instruments and for all subgroups at each timepoint using McDonald’s omega (ω) and ﻿Cronbach’s alpha (α) coefficient methods [50]. The Cronbach’s alpha estimate is considered to have been outperformed by alternative reliability measures [51], and McDonald’s omega was recommended to substitute Cronbach’s alpha in future bonding validation studies [8]. Therefore, Cronbach’s alpha is presented in this paper for comparison purposes, since a vast majority of previous studies have used this reliability method [8, 51]. According to COSMIN, Cronbach’s alpha coefficients α ≥ 0.70 fulfil internal consistency criteria for a good measurement property [49], and reliability values from alternative reliability measures are similarly interpreted [51]. PIBS item reliability statistics after the deletion of single items were also assessed.

COSMIN distinguishes between the measurement properties of an instrument and the interpretability of its scores, the latter thus not regarded as a measurement property, yet as an important instrument characteristic [31]. Descriptive statistics related to score interpretability, i.e., central tendencies (means and medians) and variability, and percentages of scale missing data, and lowest possible score [49] were calculated for the PIBS and PBQ total and subscale scores for all subgroups.

For fulfilling convergent validity of the PIBS in the clinical sample, PIBS maternal and paternal bonding scores reflecting current time would correlate strongly, as indicated by a Spearman’s rank correlation coefficient *r*_*s*_ ≥ 0.70 [31, 47, 48, 52] with scores of the PBQ total scale and the impaired bonding, and rejection and anger subscales. These two subscales are explicitly defined as constituting parts of the bonding construct, whereas the PBQ anxiety about care construct apparently lies in a broader postpartum mental health spectrum related to anxiety disorders [19, 20, 21]. For PIBS discriminant validity, it was therefore hypothesised that correlations between PIBS current scores and PBQ anxiety about care subscale scores would be *r*_*s*_ ≤ 0.60. This hypothesis was guided by the COSMIN example of hypotheses testing for construct validity, that correlations with instruments measuring related but dissimilar constructs should differ by a minimum of 0.10 from correlations with instruments measuring similar constructs [49]. It was similarly hypothesised that correlations between PIBS scores and EPDS, DASS-D, and DASS-A scores, respectively, in the community and clinical samples would be *r*_*s*_ ≤ 0.60 to demonstrate discriminant validity of the PIBS against these mental health constructs.

Further, it was hypothesised that (1) based on clinical experience, bonding scores as measured by the PIBS in both parent groups would be higher in the clinical group, indicating higher risk of bonding difficulties, than in the community group; (2) based on results from previous studies indicating that postpartum bonding improves with time from the early postpartum period [20, 28, 37], bonding scores would be higher (indicating more difficulties) in the first weeks after the child was born compared with scores at later stages in the bonding process; (3) based on previous results from studies in general population samples, bonding scores in the community group would either be similar between parent groups [27, 30] or higher in fathers than in mothers [25, 26, 28, 29], and (4) based on clinical experience in patient seeking and referral patterns to infant mental health units, bonding scores in the clinical group would be higher in the mothers than in the fathers.

Statistical analyses were performed using IBM SPSS Statistics (version 28.0.1.0; IBM Corp., Armonk, NY, USA) and *jamovi* (version 2.3) computer software retrieved from https://www.jamovi.org.

## Results

### Descriptive Characteristics of the Study Participants

Descriptive characteristics of the participants from the two population samples are presented in Table 1.

**Table 1:** Descriptive characteristics of study participants in the community (Scania Birth Cohort) and the clinical (infant mental health unit) population samples

	Community sample		Clinical sample	
	Mothers *N* = 65	Fathers *N* = 57	Mothers *N* = 100	Fathers *N* = 82
Parental age years mean (SD)	31.2 (4.0)	32.2 (5.6)	33.4 (4.9)	34.9 (5.5)
Child age months mean (SD)			12.7 (13.2)	10.9 (12.2)
At T1	1.6 (0.7)	1.7 (0.6)	–	–
At T2	6.3 (0.5)	6.5 (0.8)	–	–
At T3	12.0 (0.6)	12.1 (0.5)	–	–
Highest education *n* (%)				
Compulsory school (9 years)	2 (3.1%)	3 (5.8%)	0	3 (3.7%)
Senior high school (2–4 years)	19 (29.2%)	21 (40.4%)	19 (19.0%)	23 (28.0%)
University (≤ 3 years)	21 (32.3%)	15 (28.8%)	21 (21.0%)	14 (17.1%)
University (> 3 years)	23 (35.4%)	13 (25.0%)	54 (54.0%)	39 (47.6%)
Post-graduate education	0	0	6 (6.0%)	3 (3.7%)
Cohabitation status *n* (%)				
Living with the other parent	60 (92.3%)	56 (98.2%)	86 (86.0%)	81 (98.8%)
Living alone	3 (4.6%)	0	9 (9.0%)	1 (1.2%)
Living with their own parents	2 (3.1%)	1 (1.8%)	3 (3.0%)	0
Other living arrangement	0	0	2 (2.0%)	0
Region of birth *n* (%)				
Sweden	59 (90.8%)	51 (91.1%)	84 (84.0%)	65 (79.3%)
Scandinavia (not Sweden)	1 (1.5%)	0	3 (3.0%)	2 (2.4%)
Europe (not Scandinavia)	3 (4.6%)	3 (5.4%)	7 (7.0%)	5 (6.1%)
Outside of Europe	2 (3.1%)	2 (3.6%)	6 (6.0%)	10 (12.2%)
Information missing	0	1 (1.2%)	0	0
EPDS mean (SD**)**			–	–
At T1	6.6 (5.3)	4.1 (4.1)	–	–
At T2	6.0 (4.9)	3.6 (3.4)	–	–
At T3	5.8 (5.2)	2.7 (2.9)	–	–
DASS-D mean (SD)			6.3 (4.7)	3.3 (3.5)
At T1	2.0 (3.2)	1.4 (2.3)	–	–
At T2	1.9 (2.7)	1.2 (2.2)	–	–
At T3	2.1 (3.3)	1.1 (1.8)	–	–
DASS-A mean (SD)			4.1 (4.0)	1.9 (3.0)
At T1	1.4 (2.4)	0.8 (1.8)	–	–
At T2	1.2 (2.2)	0.7 (1.4)	–	–
At T3	1.4 (2.9)	0.9 (1.9)	–	–

*SD* standard deviation, *T1–T3* Postpartum data collection timepoints in the community sample, *EPDS* Edinburgh Postnatal Depression Scale, *DASS* Depression Anxiety Stress Scales, *DASS-D* DASS depression scale, *DASS-A* DASS anxiety scale

In the survey for the clinical sample, the parents were asked to describe the main reason why they as a family needed the treatment at the IMH unit. Of the mothers, 71% reported the main reason for needing the treatment at the IMH unit as their own difficulties, either concerning their parenting (46%) or difficulties due to mental health issues (25%), while 3% reported the main reason was their partner’s difficulties. Of the fathers, 41% reported the main reason being their own difficulties (33% concerning their parenting, 8% due to mental health issues), and 36% reported difficulties for their partner as the main reason. Other main reasons were difficulties for the child (e.g., externalising, and internalising behaviours, and feeding problems), reported as the main reason by 22% of the parents in total (not shown in Table).

### PIBS Internal Consistency, and Descriptive Statistics Related to Score Interpretability

In the community sample, the PIBS internal reliability coefficients were generally higher using the McDonald’s ω method and varied from high (ω = 0.86) to low (ω = 0.49) between timepoints (T1–T3). For the mothers they were the highest at T1 (ω = 0.85) and for the fathers at T2 (ω = 0.86). Close-to-acceptable internal consistency emerged for the fathers at T1 (ω = 0.67) and for the mothers at T3 (ω = 0.69). In the clinical sample, the PIBS internal reliability coefficients were all above 0.70 and the results were similar between the two methods McDonald’s ω and Cronbach’s α. Coefficients ranged between 0.76 and 0.89 and were somewhat higher for mothers than for fathers (Table 2).

**Table 2:** Parent-to-Infant Bonding Scale (PIBS) internal reliability coefficients, central tendencies, and variability, and percentages of scale missing data and lowest possible score in mothers and fathers in the community and clinical population samples

PIBS version	Community sample (Scania Birth Cohort)												Clinical sample (infant mental health unit)							
	PIBS first weeks				PIBS current				PIBS current				PIBS first weeks				PIBS current			
	T1				T2				T3											
Child age	1 month				6 months				12 months				1–50 months^a^							
Parent group	Mothers		Fathers		Mothers		Fathers		Mothers		Fathers		Mothers		Fathers		Mothers		Fathers	
No. of participants, *N*^b^	*N*=61		*N*=54		*N*=56		*N*=49		*N*=35		*N*=33		Mothers *N*=100, Fathers *N*=82							
Internal reliability coefficient	ω	α	ω	α	ω	α	ω	α	ω	α	ω	α	ω	α	ω	α	ω	α	ω	α
	0.85	0.79	0.67	0.54	0.49	0.51	0.86	0.79	0.69	0.59	0.62	0.43	0.89	0.89	0.82	0.81	0.85	0.84	0.79	0.76
Mean (SD)	1.3 (2.4)		1.3 (1.6)		0.4 (0.8)		0.8 (1.7)		0.6 (1.2)		0.7 (1.0)		5.6 (5.5)		3.7 (4.1)		3.3 (3.5)		2.2 (2.5)	
Median (quartiles: Q1, Q3)	0 (0, 1.75)		1 (0, 3)		0 (0, 0)		0 (0, 1)		0 (0, 1)		0 (0, 1)		4 (1, 7.75)		2 (0, 6)		2 (1, 5)		1 (0, 4)	
No. of responding^c^; % missing	60; 1.6%		52; 3.7%		55; 1.8%		48; 2.0%		35; 0%		32; 3.0%		92; 8.0%		79; 3.7%		99; 1.0%		80; 2.5%	
Percent lowest possible score	58.3%		46.2%		80.0%		60.4%		74.3%		59.4%		18.5%		25.3%		24.2%		33.8%	

*PIBS* Parent-to-Infant Bonding Scale, *PIBS first weeks* reflect bonding in the first weeks (retrospectively), *PIBS current* reflect bonding in current time, *T1–T3* Postpartum data collection timepoints for the community sample, *α* Cronbach’s alpha coefficient, *ω* McDonald’s omega coefficient, *% missing* percentage of scale missing data

^a^Mean child age in the clinical sample: mothers: 12.7 months; fathers: 10.9 months

^b^*N* = No. of participants who filled in the questionnaire

^c^No. of respondents with complete PIBS data

There were no consistent patterns of internal reliability coefficients being significantly altered after the deletion of single items in either parent or population group. For example, deleting single items for the community mothers at T1 yielded McDonald’s ω coefficients ranging between ω = 0.81 and ω = 0.86. Specifically, deleting the item “Dissatisfied” (*Olust* in Swedish) decreased the coefficient from ω = 0.85 to ω = 0.81, while deleting either “Resentful” (*Motvilja*) or “Protective” (*Beskyddande*) increased it from ω = 0.85 to ω = 0.86 (not shown in Table).

PIBS floor effects, i.e., more than 15% of participants with the lowest possible score [52] were evident in both parent groups in both samples, with higher percentages of lowest possible score in the community compared to the clinical sample (Table 2).

### Internal Consistency of the Comparator Instruments, and PBQ Descriptive Statistics Related to Score Interpretability

McDonald’s ω coefficients for the PBQ total scale and subscales in the clinical sample were 0.70 or above except for the anxiety about care subscale (ω = 0.61 in mothers and ω = 0.66 in fathers). Percentages of lowest possible PBQ total or subscale score in mothers and fathers in the clinical sample were all < 15% (Table 3).

**Table 3:** Postpartum Bonding Questionnaire (PBQ) internal reliability coefficients, central tendencies, and variability, and percentages of scale missing data and lowest possible score in the clinical sample

PBQ total or subscale	PBQ total		PBQ IB		PBQ RA		PBQ AC		PBQ total		PBQ IB		PBQ RA		PBQ AC	
Parent group	Mothers								Fathers							
Internal reliability coefficient	ω	α	ω	α	ω	α	ω	α	ω	α	ω	α	ω	α	ω	α
	0.91	0.90	0.86	0.85	0.81	0.80	0.61	0.59	0.89	0.89	0.83	0.82	0.70	0.68	0.66	0.62
Mean (SD)	24.4 (13.5)		13.1 (7.5)		6.1 (4.6)		5.2 (3.2)		17.7 (11.7)		9.8 (6.6)		4.5 (3.5)		3.5 (2.9)	
Median (quartiles: Q1, Q3)	23 (14.75, 31)		13 (7, 17)		5 (3, 9)		9 (3, 7)		15 (8.5, 24)		8.5 (5, 13.25)		4 (1, 7)		2 (1, 5)	
No. of responding^a^; % missing	90; 10%		91; 9.0%		97; 3.0%		100; 0%		77; 6.1%		78; 4.9%		79; 3.7%		81; 1.2%	
Percent lowest possible score	1.1%		1.1%		8.2%		2.0%		3.9%		2.6%		12.7%		9.9%	

Mothers *N* = 100, Fathers *N* = 82

*PBQ total* Postpartum Bonding Questionnaire total scale (23 items), *IB* impaired bonding (12 items), *RA* rejection and anger (7 items), *AC* anxiety about care (4 items), *α* Cronbach’s alpha coefficient, *ω* McDonald’s omega coefficient, *% missing* percentage of scale missing data

^a^No. of respondents with complete PBQ data

In the community sample at T1–T3, all internal reliability coefficients (McDonald’s ω and ﻿Cronbach’s α) of the EPDS, DASS-D, and DASS-A were above 0.70, ranging between 0.73 and 0.93. In the clinical sample, the DASS-D and DASS-A internal reliability coefficients ranged between 0.83 and 0.89 (not shown in Table).

### PIBS Correlations in the Clinical Population Sample

The PIBS current scores correlated strongly with the PBQ total scores, and with scores of the PBQ impaired bonding, and rejection and anger subscales in both mothers and fathers, *r*_*s*_ = 0.74 to *r*_*s*_ = 0.80, all *p* < 0.001. The correlations between the PIBS and PBQ anxiety about care scores were lower, *r*_*s*_ = 0.35 for mothers, and* r*_*s*_ = 0.49 for fathers, both *p* < 0.001 (Table 4).

**Table 4:** Correlations between the Parent-to-Infant Bonding Scale (PIBS) and Postpartum Bonding Questionnaire (PBQ) total and subscale scores in the clinical sample

	PIBS current	PBQ total	PBQ IB	PBQ RA	PBQ AC
PIBS current	–	0.76***	0.74***	0.80***	0.49***
PBQ total	0.76***	–	0.95***	0.88***	0.80***
PBQ IB	0.76***	0.95***	–	0.78***	0.67***
PBQ RA	0.76***	0.92***	0.82***	–	0.57***
PBQ AC	0.35***	0.62***	0.46***	0.45***	–

Mothers *N* = 100, Fathers *N* = 82. The mothers’ correlation coefficients are displayed below the diagonal. The fathers’ correlation coefficients are displayed above the diagonal

*PIBS current* Parent-to-Infant Bonding Scale reflecting bonding in current time, *PBQ total* Postpartum Bonding Questionnaire total scale (23 items), *IB* impaired bonding (12 items), *RA* rejection and anger (7 items), *AC* anxiety about care (4 items)

***p < 0.001

The correlations between PIBS first weeks and PIBS current scores in the clinical sample were *r*_*s*_ = 0.51 for mothers, and *r*_*s*_ = 0.66 for fathers, both *p* < 0.001 (not shown in Table).

### PIBS Correlations with the EPDS, DASS-D, and DASS-A

In the community and clinical samples, the correlations between PIBS scores and EPDS, DASS-D, and DASS-A scores, respectively, for mothers and fathers were all less than 0.60 (Table 5).

**Table 5:** Correlations between Parent-to-Infant Bonding Scale (PIBS) scores and mental health measurements scores in the community and clinical samples

Child age	Community sample												Clinical sample		
	1 month Mothers *N* = 61, Fathers *N* = 54				6 months Mothers *N* = 56, Fathers *N* = 49				12 months Mothers *N* = 35, Fathers *N* = 33				1–50 months^a^ Mothers *N* = 100, Fathers *N* = 82		
	PIBS	EPDS	DASS-D	DASS-A	PIBS	EPDS	DASS-D	DASS-A	PIBS	EPDS	DASS-D	DASS-A	PIBS	DASS-D	DASS-A
PIBS	–	0.46***	0.37*	0.20	–	0.31*	0.29	0.23	–	0.10	0.22	0.05	–	0.44***	0.36**
EPDS	0.25	–	0.65***	0.44**	0.17	–	0.65***	0.64***	0.10	–	0.65***	0.36*	n/a	n/a	n/a
DASS-D	0.37**	0.76***	–	0.52***	0.19	0.74***	–	0.55***	0.31	0.77***	–	0.24	0.54***	–	0.45***
DASS-A	0.28*	0.64***	0.66***	–	0.04	0.58***	0.43***	–	0.25	0.41***	0.55***	–	0.27**	0.67***	–

Correlation coefficients for mothers and fathers are displayed below and above the diagonals, respectively. Community sample 1 month PIBS scores reflect bonding in the first weeks, all other PIBS scores reflect current time. Mothers’ correlation coefficients are displayed below the diagonals. Fathers’ correlation coefficients are displayed above the diagonals

*PIBS* Parent-to-Infant Bonding Scale, *EPDS* Edinburgh Postnatal Depression Scale, *DASS* Depression Anxiety Stress Scales, *DASS-D* DASS depression scale, *DASS-A* DASS anxiety scale, *n/a* not applicable

*p < 0.05; **p < 0.01; ***p < 0.001

^a^Mean child age for the mothers: 12.7 months; mean child age for the fathers: 10.9 months

### Comparisons of PIBS Scores Between Groups and Between Timepoints Within Groups

The median PIBS first weeks as well as current scores in the clinical sample were higher, indicating a higher risk of bonding difficulties, than PIBS scores in the community sample (T1–T3) for both mothers and fathers (Table 2), with medium to large effect sizes (*r*), *U* = 1326.50,* z* = -3.51,* p* < 0.001,* r* = 0.31 to *U* = 999.50,* z* = -6.81,* p* < 0.001, *r* = 0.55.

In the community sample, median PIBS scores were higher at T1 compared with scores at T2 and T3 in both mothers and fathers (Table 2) with small to medium effect sizes (*ω*^*2*^), *F*(1.7, 52.6) = 3.61, *p* = 0.041*, ω*^*2*^ = 0.05, and *F*(1.8, 48.3) = 3.48*, p* = 0.044, *ω*^*2*^ = 0.038 respectively. Planned contrasts (T1 vs. T2; T2 vs. T3), revealed there was no significant difference between T2 and T3, but there was a significant reduction in PIBS scores between T1 and T2, *F*(1, 31) = 5.42,* p* = 0.027 for the mothers, and *F*(1, 27) = 6.24,* p* = 0.019 for the fathers.

Furthermore, in the community sample at T2 the fathers’ PIBS scores were significantly higher than the mothers’ scores (Table 2), *U* = 1031.00, *z* = − 2.24, *p* = 0.025, *r* = 0.22, whereas the PIBS scores at T1 and T3 did not differ significantly between the parent groups (Table 2).

In the clinical sample, the PIBS first weeks scores, retrospectively reflecting bonding in the first weeks, were higher than the PIBS current scores in both mothers and fathers (Table 2), with medium effect sizes, *T* = 571.5,* p* < 0.001,* r* = 0.47, and *T* = 314.0, *p* < 0.001,* r* = 0.42 respectively.

Further, the mothers in the clinical population reported higher PIBS first weeks, as well as current scores than the fathers (Table 2), with small effect sizes, *U* = 2899.50, *z* = − 2.29, *p* = 0.022, *r* = 0.18, and *U* = 3283.00, *z* = − 2.00, *p* = 0.046, *r* = 0.15 respectively.

## Discussion

This study was designed to develop and validate a new bonding instrument, the Parent-to-Infant Bonding Scale (PIBS), to enable the inclusion of all parents in future evaluations of parent-infant bonding affecting child life-course health development. Therefore, the measurement properties internal consistency and construct validity of the PIBS were assessed for mothers and fathers in clinical and community population samples. Aspects of PIBS score interpretability were also investigated. It was beyond the scope of this paper to explore aspects of clinical utility of the PIBS such as for screening purposes or evaluation of treatment needs and outcomes, so these aspects would need to be addressed in future studies.

In the clinical sample in both parent groups, good internal consistency emerged demonstrating the interrelatedness of the PIBS items, and convergent and discriminant validities of the PIBS were apparent. Discriminant validity of the PIBS against the mental health constructs of depressive symptoms and anxiety was demonstrated also in the community group. Comparisons of bonding scores between subgroups (mothers, fathers, community, clinical) and between timepoints were in accordance with the hypotheses, corroborating PIBS construct validity. These findings also appear to support PIBS interpretability in terms of detecting minimal important differences [31, 47–49].

In the community sample, PIBS internal reliability coefficients varied between the postpartum timepoints. For the fathers, the internal consistency was close-to-acceptable in the early postpartum period and good when the child was 6 months old, whereas good internal consistency emerged for the mothers in the early postpartum period but not when the child was 6 months old. Regarding the latter results for the mothers, a previous study [37] showed similar patterns for three bonding measurements, including the PBQ and MIBS, used with new mothers in a general population sample. A probable explanation for these findings is that the reliability coefficient value varies with the level of variability [53]. This relationship can be observed in the van Bussel et al. [37] study and seems apparent also in the current study, i.e., the lower the variability the lower the internal reliability estimates.

Since the prevalence of postpartum bonding disorders in the general population is expected to be low, estimated to be approximately 1% [7], the findings in the community sample of low PIBS scores, pronounced floor effects, and subsequent low variability, all of which are related to score interpretability, were expected. However, PIBS floor effects appeared also in the clinical sample despite higher scores in both parent groups, whereas no floor effects were observed for the PBQ in that sample. One factor explaining these differing results between the bonding instruments may be the number of response alternatives, with the PIBS and the PBQ consisting of four and six alternatives, respectively. Another factor may be that some PBQ items are formulated such that they are easier for parents to agree with compared to the PIBS. For example, the PBQ item “My baby irritates me” would seem easier to agree with than endorsing that you feel “Aggressive” towards your child, as stated in the PIBS. Further development of the PIBS concerning items and response alternatives may enhance its score interpretability further. Such development could also alleviate potential issues with internal reliability statistics related to the level of variability, as discussed above.

Although the PIBS is a different instrument, it was developed from the MIBS. Therefore, comparisons with the S-MIBS [54], a Swedish translation of the MIBS not published at the start of this study, could be of interest. The validation of the S-MIBS was performed at a single timepoint with community mothers of infants aged 6–13 weeks. The PIBS measurement properties presented here would seem somewhat stronger. For example, the comparable internal reliability estimate (Cronbach’s alpha) for the community mothers at the comparable child age (T1) was higher for the PIBS than for S-MIBS (α = 0.79 and α = 0.68, respectively). It should also be noted that their finding that the removal of the item “Protective” significantly increased the scale’s internal consistency [54] was not apparent in the present community nor clinical sample.

### Strengths and Limitations

A strength of the study was validating the PIBS in four subgroups (mothers and fathers from two different types of population). Furthermore, a repeated measures design was used for the community sample covering validation of the PIBS during different developmental time periods, i.e., the early, middle, and late postpartum periods. Another strength was assessing internal consistency using McDonald’s omega [8, 51]. Further, information was reported to evaluate aspects of score interpretability which is generally missing in bonding measurement validation studies [8], adding to the strengths of this study. A study limitation was the small sample sizes, particularly in the community sample at T3 which is why those results should be interpreted with caution. Structural validity assessments using factor analysis were not performed due to the COSMIN sample size requirements for this type of analysis [52]. Due to the requests from the ethics committee, the convergent validity of the PIBS against the PBQ could not be assessed in the community sample. In the clinical sample, the PIBS first weeks measure was used for some of the hypotheses testing (not the convergent and discriminant validity testing for which the PIBS current measure was used). The early bonding was thus in the sample assessed retrospectively up to four years after the birth of the child. There is evidence that the immediate postpartum period and the early feelings towards the child are well remembered by mothers one year later [55]. However, it has not been shown for longer periods and not for fathers, which is why the accuracy of this estimate may be treated with caution.

### Implications for Future Research and Clinical Practice

This work contributes to enabling research on life-course health development for children as related to parental bonding during different developmental time periods in maternal and paternal community and clinical populations. The similarities in the measurement properties of the PIBS between the mothers and fathers indicate its value for future studies of the unique impacts of maternal and paternal bonding on the offspring through longitudinal studies using cohorts from general and clinical populations. Such studies may well include investigations of the potential effects of interactions between maternal and paternal bonding on child outcomes. The work also contributes to potential future clinical use of the instrument helping health professionals support the parent–child relationship for possible long-term benefits for the whole family. Further validation of the PIBS could concern other developmental time periods including pregnancy and older child age groups, content validity assessing relevance and comprehensiveness of the PIBS items for fathers, and measurement invariance to determine whether the PIBS items function similarly in different subgroups. Assessing the criterion validity of the PIBS against a structured clinical interview, for example the Stafford Interview which was developed for mothers during pregnancy and the postpartum period in community and clinical settings [56], is desirable.

## Summary

To incorporate both parents as agents in the important early development of their infants, and in future evaluations of how parent-infant bonding affects the offspring from a life-course perspective in community as well as in clinical populations, a self-report questionnaire for the measurement of parental bonding was developed. Overall, the measurement properties of the PIBS support its validity as a measure of maternal and paternal bonding in both types of population. Guidance for potential future development of the instrument regarding aspects related to score interpretability was given. The similarities in the measurement properties between mothers and fathers indicate the value of the PIBS for comparing aspects of bonding between these parent groups in general and clinical populations.

## Supplementary Information

Below is the link to the electronic supplementary material.

Supplementary file1 (DOCX 50 kb)

## Data Availability

No datasets were generated or analysed during the current study.

## References

[1] Burlingham M, Maguire L, Hibberd L, Turville N, Cowdell F, Bailey E (2024) The needs of multiple birth families during the first 1001 critical days: a rapid review with a systematic literature search and narrative synthesis. Public Health Nurs 41(1):112–126. 10.1111/phn.13259 37916962 10.1111/phn.13259

[2] Zijlstra A, Joosten D, van Nieuwenhuijzen M, de Castro BO (2023) The first 1001 days: a scoping review of parenting interventions strengthening good enough parenting in parents with intellectual disabilities. J Intellect Disabil. 10.1177/17446295231219301 38050742 10.1177/17446295231219301 PMC12084667

[3] Black MM, Walker SP, Fernald LCH, Andersen CT, DiGirolamo AM, Lu C, McCoy DC, Fink G, Shawar YR, Shiffman J, Devercelli AE, Wodon QT, Vargas-Barón E, Grantham-McGregor S, Committee LECDSS (2017) Early childhood development coming of age: science through the life course. Lancet 389(10064):77–90. 10.1016/S0140-6736(16)31389-7 27717614 10.1016/S0140-6736(16)31389-7 PMC5884058

[4] Phua DY, Kee MZL, Meaney MJ (2020) Positive maternal mental health, parenting, and child development. Biol Psychiat 87(4):328–337. 10.1016/j.biopsych.2019.09.028 31839213 10.1016/j.biopsych.2019.09.028

[5] Fuertes M, Ribeiro C, Gonçalves JL, Rodrigues C, Beeghly M, Lopes-Dos-Santos P, Lamônica DAC (2020) Maternal perinatal representations and their associations with mother-infant interaction and attachment: a longitudinal comparison of Portuguese and Brazilian dyads. Int J Psychol 55(2):224–233. 10.1002/ijop.12577 30847895 10.1002/ijop.12577

[6] Le Bas GA, Youssef GJ, Macdonald JA, Rossen L, Teague SJ, Kothe EJ, McIntosh JE, Olsson CA, Hutchinson DM (2020) The role of antenatal and postnatal maternal bonding in infant development: a systematic review and meta-analysis. Soc Dev 29(1):3–20. 10.1111/sode.12392

[7] Brockington IF (2016) Emotional rejection of the infant: status of the concept. Psychopathology 49(4):247–260. 10.1159/000448334 27583348 10.1159/000448334

[8] Wittkowski A, Vatter S, Muhinyi A, Garrett C, Henderson M (2020) Measuring bonding or attachment in the parent-infant-relationship: a systematic review of parent-report assessment measures, their psychometric properties and clinical utility. Clin Psychol Rev 82:101906. 10.1016/j.cpr.2020.101906 32977111 10.1016/j.cpr.2020.101906 PMC7695805

[9] Nakić Radoš S, Hairston I, Handelzalts JE (2023) The concept analysis of parent-infant bonding during pregnancy and infancy: a systematic review and meta-synthesis. J Reproduct Infant Psychol. 10.1080/02646838.2022.2162487 10.1080/02646838.2022.2162487 36588501

[10] Suzuki D, Ohashi Y, Shinohara E, Usui Y, Yamada F, Yamaji N, Sasayama K, Suzuki H, Nieva RF, da Silva Lopes K, Miyazawa J, Hase M, Kabashima M, Ota E (2022) The current concept of paternal bonding: a systematic scoping review. Healthcare (Basel) 10(11):2265. 10.3390/healthcare10112265 36421589 10.3390/healthcare10112265 PMC9690989

[11] Hashijiri K, Watanabe Y, Fukui N, Motegi T, Ogawa M, Egawa J, Enomoto T, Someya T (2021) Identification of bonding difficulties in the peripartum period using the mother-to-infant bonding scale-Japanese version and its tentative cutoff points. Neuropsychiatr Dis Treat 17:3407–3413. 10.2147/ndt.S336819 34848961 10.2147/NDT.S336819 PMC8616728

[12] Altaweli R, Roberts J (2010) Maternal-infant bonding: a concept analysis. Br J Midwifery 18(9):552–559. 10.12968/bjom.2010.18.9.78062

[13] Bicking Kinsey C, Hupcey JE (2013) State of the science of maternal-infant bonding: a principle-based concept analysis. Midwifery 29(12):1314–1320. 10.1016/j.midw.2012.12.019 23452661 10.1016/j.midw.2012.12.019 PMC3838467

[14] Suryaningsih EK, Gau ML, Wantonoro W (2020) Concept analysis of maternal-fetal attachment. Belitung Nurs J 6(5):157–164. 10.33546/bnj.1194

[15] Trombetta T, Giordano M, Santoniccolo F, Vismara L, Della Vedova AM, Rollè L (2021) Pre-natal attachment and parent-to-infant attachment: a systematic review. Front Psychol 12:620942. 10.3389/fpsyg.2021.620942 33815204 10.3389/fpsyg.2021.620942 PMC8011495

[16] Hill R, Flanagan J (2020) The maternal-infant bond: clarifying the concept. Int J Nurs Knowl 31(1):14–18. 10.1111/2047-3095.12235 30623601 10.1111/2047-3095.12235

[17] Ainsworth MD (1979) Infant-mother attachment. Am Psychol 34(10):932–937. 10.1037/0003-066x.34.10.932 517843 10.1037//0003-066x.34.10.932

[18] Bowlby J (1969) Attachment and loss, vol 1. Attachment, Pimlico

[19] Brockington IF, Fraser C, Wilson D (2006) The postpartum bonding questionnaire: a validation. Arch Women’s Ment Health 9(5):233–242. 10.1007/s00737-006-0132-1 16673041 10.1007/s00737-006-0132-1

[20] Taylor A, Atkins R, Kumar R, Adams D, Glover V (2005) A new mother-to-infant bonding scale: links with early maternal mood. Arch Women’s Ment Health 8(1):45–51. 10.1007/s00737-005-0074-z 15868385 10.1007/s00737-005-0074-z

[21] Brockington IF, Butterworth R, Glangeaud-Freudenthal N (2017) An international position paper on mother-infant (perinatal) mental health, with guidelines for clinical practice. Arch Women’s Ment Health 20(1):113–120. 10.1007/s00737-016-0684-7 27826750 10.1007/s00737-016-0684-7 PMC5237446

[22] Anderson A (1996) The father-infant relationship: becoming connected. J Soc Pediatr Nurs 1(2):83–92. 10.1111/j.1744-6155.1996.tb00005.x 8933480 10.1111/j.1744-6155.1996.tb00005.x

[23] de Waal N, van den Heuvel MI, Nyklíček I, Pop VJM, Boekhorst M (2023) Paternal bonding in pregnancy and early parenthood: a qualitative study in first-time fathers. J Reproduct Infant Psychol. 10.1080/02646838.2023.2252890 10.1080/02646838.2023.2252890 37650726

[24] Abraham E, Feldman R (2018) The neurobiology of human allomaternal care; implications for fathering, coparenting, and children’s social development. Physiol Behav 193:25–34. 10.1016/j.physbeh.2017.12.034 29730038 10.1016/j.physbeh.2017.12.034

[25] de Cock ES, Henrichs J, Vreeswijk CM, Maas AJ, Rijk CH, van Bakel HJ (2016) Continuous feelings of love? The parental bond from pregnancy to toddlerhood. J Fam Psychol 30(1):125–134. 10.1037/fam0000138 26280095 10.1037/fam0000138

[26] Edhborg M, Matthiesen AS, Lundh W, Widström AM (2005) Some early indicators for depressive symptoms and bonding 2 months postpartum—a study of new mothers and fathers. Arch Women’s Ment Health 8(4):221–231. 10.1007/s00737-005-0097-5 16172838 10.1007/s00737-005-0097-5

[27] Figueiredo B, Costa R, Pacheco A, Pais A (2007) Mother-to-infant and father-to-infant initial emotional involvement. Early Child Dev Care 177(5):521–532. 10.1080/03004430600577562

[28] Hall RA, Hoffenkamp HN, Tooten A, Braeken J, Vingerhoets AJ, Van Bakel HJ (2015) Child-rearing history and emotional bonding in parents of preterm and full-term infants. J Child Fam Stud 24:1715–1726. 10.1007/s10826-014-9975-7

[29] Kerstis B, Aarts C, Tillman C, Persson H, Engström G, Edlund B, Öhrvik J, Sylvén S, Skalkidou A (2016) Association between parental depressive symptoms and impaired bonding with the infant. Arch Women’s Ment Health 19(1):87–94. 10.1007/s00737-015-0522-3 25854998 10.1007/s00737-015-0522-3

[30] Nakić RS (2021) Parental sensitivity and responsiveness as mediators between postpartum mental health and bonding in mothers and fathers. Front Psych 12:723418. 10.3389/fpsyt.2021.723418 10.3389/fpsyt.2021.723418 PMC8440918 34539469

[31] Mokkink LB, Terwee CB, Knol DL, Stratford PW, Alonso J, Patrick DL, Bouter LM, de Vet HCW (2010) The COSMIN checklist for evaluating the methodological quality of studies on measurement properties: a clarification of its content. BMC Med Res Methodol 10(1):1–8. 10.1186/1471-2288-10-22 20298572 10.1186/1471-2288-10-22 PMC2848183

[32] Brockington IF, Oates J, George S, Turner D, Vostanis P, Sullivan M, Loh C, Murdoch C (2001) A screening questionnaire for mother-infant bonding disorders. Arch Women’s Ment Health 3:133–140. 10.1007/s007370170010

[33] Tedgård E, Tedgård U, Råstam M, Johansson BA (2020) Parenting stress and its correlates in an infant mental health unit: a cross-sectional study. Nord J Psychiatry 74(1):30–39. 10.1080/08039488.2019.1667428 31553257 10.1080/08039488.2019.1667428

[34] O’Higgins M, Roberts IS, Glover V, Taylor A (2013) Mother-child bonding at 1 year; associations with symptoms of postnatal depression and bonding in the first few weeks. Arch Women’s Ment Health 16(5):381–389. 10.1007/s00737-013-0354-y 23604546 10.1007/s00737-013-0354-y

[35] Kumar RC (1997) “Anybody’s child”: severe disorders of mother-to-infant bonding. Br J Psychiatry 171:175–181. 10.1192/bjp.171.2.175 9337956 10.1192/bjp.171.2.175

[36] Demir E, Öz S, Aral N, Gürsoy F (2024) A reliability generalization meta-analysis of the mother-to-infant bonding scale. Psychol Rep 127(1):447–464. 10.1177/00332941221114413 35815798 10.1177/00332941221114413

[37] van Bussel JC, Spitz B, Demyttenaere K (2010) Three self-report questionnaires of the early mother-to-infant bond: reliability and validity of the Dutch version of the MPAS, PBQ and MIBS. Arch Women’s Ment Health 13(5):373–384. 10.1007/s00737-009-0140-z 20127128 10.1007/s00737-009-0140-z

[38] Wittkowski A, Wieck A, Mann S (2007) An evaluation of two bonding questionnaires: a comparison of the mother-to-infant bonding scale with the postpartum bonding questionnaire in a sample of primiparous mothers. Arch Women’s Ment Health 10:171–175. 10.1007/s00737-007-0191-y 17607505 10.1007/s00737-007-0191-y

[39] Cortina JM (1993) What is coefficient alpha? An examination of theory and applications. J Appl Psychol 78(1):98. 10.1037/0021-9010.78.1.98

[40] Alfonsson S, Wallin E, Maathz P (2017) Factor structure and validity of the depression, anxiety and stress scale-21 in Swedish translation. J Psychiatr Ment Health Nurs 24(2–3):154–162. 10.1111/jpm.12363 28124410 10.1111/jpm.12363

[41] Lovibond PF, Lovibond SH (1995) The structure of negative emotional states: comparison of the depression anxiety stress scales (DASS) with the beck depression and anxiety inventories. Behav Res Ther 33(3):335–343. 10.1016/0005-7967(94)00075-U 7726811 10.1016/0005-7967(94)00075-u

[42] Cox JL, Holden JM, Sagovsky R (1987) Detection of postnatal depression. Development of the 10-item Edinburgh Postnatal depression scale. Br J Psychiatry 150:782–786. 10.1192/bjp.150.6.782 3651732 10.1192/bjp.150.6.782

[43] Lundh W, Gyllang C (1993) Use of the Edinburgh postnatal depression scale in some Swedish child health care centres. Scand J Caring Sci 7(3):149–154. 10.1111/j.1471- 8108616 10.1111/j.1471-6712.1993.tb00190.x

[44] Sambrook Smith M, Cairns L, Pullen LSW, Opondo C, Fellmeth G, Alderdice F (2022) Validated tools to identify common mental disorders in the perinatal period: a systematic review of systematic reviews. J Affect Disord 298(Pt A):634–643. 10.1016/j.jad.2021.11.011 34763033 10.1016/j.jad.2021.11.011

[45] Berg RC, Solberg BL, Glavin K, Olsvold N (2022) Instruments to identify symptoms of paternal depression during pregnancy and the first postpartum year: a systematic scoping review. Am J Men’s Health 16(5):1–16. 10.1177/15579883221114984 10.1177/15579883221114984 PMC9490477 36124356

[46] Mokkink LB, de Vet HCW, Prinsen CAC, Patrick DL, Alonso J, Bouter LM, Terwee CB (2018) COSMIN risk of bias checklist for systematic reviews of patient-reported outcome measures. Qual Life Res 27(5):1171–1179. 10.1007/s11136-017-1765-4 29260445 10.1007/s11136-017-1765-4 PMC5891552

[47] Mokkink LB, Terwee CB, Patrick DL, Alonso J, Stratford PW, Knol DL, Bouter LM, de Vet HCW (2010) The COSMIN checklist for assessing the methodological quality of studies on measurement properties of health status measurement instruments: an international Delphi study. Qual Life Res 19:539–549. 10.1007/s11136-010-9606-8 20169472 10.1007/s11136-010-9606-8 PMC2852520

[48] Mokkink LB, Terwee CB, Patrick DL, Alonso J, Stratford PW, Knol DL, Bouter LM, de Vet HCW (2010) The COSMIN study reached international consensus on taxonomy, terminology, and definitions of measurement properties for health-related patient-reported outcomes. J Clin Epidemiol 63(7):737–745. 10.1016/j.jclinepi.2010.02.006 20494804 10.1016/j.jclinepi.2010.02.006

[49] Prinsen CA, Mokkink LB, Bouter LM, Alonso J, Patrick DL, De Vet HC, Terwee CB (2018) COSMIN guideline for systematic reviews of patient-reported outcome measures. Qual Life Res 27:1147–1157. 10.1007/s11136-018-1798-3 29435801 10.1007/s11136-018-1798-3 PMC5891568

[50] Zinbarg RE, Revelle W, Yovel I, Li W (2005) Cronbach’s α, Revelle’s β, and McDonald’s ω H: their relations with each other and two alternative conceptualizations of reliability. Psychometrika 70(1):123–133. 10.1007/s11336-003-0974-7

[51] McNeish D (2018) Thanks coefficient alpha, we’ll take it from here. Psychol Methods 23(3):412. 10.1037/met0000144 28557467 10.1037/met0000144

[52] Terwee CB, Bot SD, de Boer MR, van der Windt DA, Knol DL, Dekker J, Bouter LM, de Vet HCW (2007) Quality criteria were proposed for measurement properties of health status questionnaires. J Clin Epidemiol 60(1):34–42. 10.1016/j.jclinepi.2006.03.012 17161752 10.1016/j.jclinepi.2006.03.012

[53] Amirrudin M, Nasution K, Supahar S (2021) Effect of variability on Cronbach alpha reliability in research practice. J Matematika Stat Komputasi 17(2):223–230. 10.20956/jmsk.v17i2.11655

[54] Mörelius E, Elander A, Saghamre E (2021) A Swedish translation and validation of the mother-to-infant bonding scale. Scand J Public Health 49(4):465–470. 10.1177/1403494820910336 32156193 10.1177/1403494820910336

[55] Robson KM, Kumar R (1980) Delayed onset of maternal affection after childbirth. Br J Psychiatry 136:347–353. 10.1192/bjp.136.4.347 7388242 10.1192/bjp.136.4.347

[56] Brockington IF, Chandra P, Bramante A, Dubow H, Fakhers W, Garcia-Esteve L, Hofberg K, Moussas S, Palcios-Hernándes B, Parfitts Y, Shieh P (2017) The Stafford interview. A comprehensive interview for mother-infant psychiatry. Arch Women’s Ment Health 20(1):107–112. 10.1007/s00737-016-0683-8 27778149 10.1007/s00737-016-0683-8 PMC5237445

[57] World Medical Association (2013) World Medical Association declaration of Helsinki: ethical principles for medical research involving human subjects. JAMA 310(20):2191–2194. 10.1001/jama.2013.281053 24141714 10.1001/jama.2013.281053

