# Supplementary material for: Development of the Parent-to-Infant Bonding Scale: Validation in Swedish Mothers and Fathers in Community and Clinical Contexts
Source: Child Psychiatry Hum Dev. 2024 May 17;57(2):324–36. doi: 10.1007/s10578-024-01699-x (PMC13128690; doi:10.1007/s10578-024-01699-x)
Supplement: Supplementary file 1 — Supplementary file1 (DOCX 50 kb) [file 10578_2024_1699_MOESM1_ESM.docx]

Supplementary material

**Development of the Parent-to-Infant Bonding Scale: Validation in Swedish Mothers and Fathers in Community and Clinical Contexts**

Child Psychiatry & Human Development

Sara Lindeberg, Eva Tedgård, Birgitta Kerstis, Ulf Tedgård, Alyx Taylor, Peter Jönsson

DOI 10.1007/s10578-024-01699-x

**Corresponding author:** Sara Lindeberg MD, PhD, Lund University, Faculty of medicine, Department of Health Sciences, Child and Family Health, Lund, Sweden. E-mail: [sara.lindeberg@med.lu.se](mailto:sara.lindeberg@med.lu.se).

**Nedan ser du ett antal exempel på känslor som man kan ha för sitt barn under de första veckorna**

**efter det att barnet föddes.** Sätt för varje exempel ett kryss i den ruta som bäst beskriver hur du

kände för ditt barn under de allra första veckorna.

*or*

**Nedan ser du ett antal exempel på känslor som man kan ha för sitt barn.** Sätt för varje exempel

ett kryss i den ruta som bäst beskriver hur du känner för ditt barn nu.

|  | Stämmer väldigt bra | Stämmer  bra | Stämmer lite grann | Stämmer  inte alls |
| --- | --- | --- | --- | --- |
| Kärleksfull |  |  |  |  |
| Olust |  |  |  |  |
| Neutral eller kände ingenting |  |  |  |  |
| Glädjefylld |  |  |  |  |
| Ogillande |  |  |  |  |
| Motvilja |  |  |  |  |
| Beskyddande |  |  |  |  |
| Besviken |  |  |  |  |
| Aggressiv |  |  |  |  |

***PIBS*** *first weeks*

***PIBS*** *current*

**SI 1** The Swedish *Parent-to-Infant Bonding Scale*, PIBS, *first weeks* and *current*

| **Original PBQ** | **Swedish PBQ** | **Back-translation** |
| --- | --- | --- |
| I feel close to my baby (IB) | Jag känner mig nära mitt barn | I feel close to my child |
| I wish the old days when I had no baby would come back (IB) | Jag önskar att de dagar då jag inte hade barn kunde komma tillbaka | I wish that I could go back to the days before I had a child *(I wish that the days from before I had a child would come back)* |
| I feel distant from my baby (RA) | Jag är avståndstagande till mitt barn | I feel distant from my child |
| I love to cuddle my baby (RA) | Jag älskar att gosa med mitt barn | I like *(love)* to snuggle with my child |
| I regret having this baby (RA) | Jag ångrar att jag fick det här barnet | I regret having this child |
| The baby does not seem to be mine (IB) | Barnet känns inte som mitt | My *(This)* child doesn’t feel like one of my own |
| My baby winds me up (IB) | Barnet stressar upp mig | My *(This)* child stresses me out |
| I love my baby to bits (IB) | Jag älskar mitt barn jättemycket | I love my child very much |
| I feel happy when my baby smiles or laughs (IB) | Jag blir lycklig när mitt barn ler eller skrattar | It makes me feel happy to see my child  smile or laugh |
| My baby irritates me (IB) | Mitt barn irriterar mig | My child irritates me |
| I enjoy playing with my baby (RA) | Jag tycker om att leka med mitt barn | I enjoy playing with my child |
| My baby cries too much (IB) | Mitt barn gråter för mycket | My child cries too much |
| I feel trapped as a mother (IB) | Jag känner mig instängd som förälder | I feel trapped as a parent |
| I feel angry with my baby (RA) | Jag känner mig arg på mitt barn | I feel angry at my child |
| I resent my baby (IB) | Jag känner motvilja mot mitt barn | I detest *(resent, loathe)* my child |
| My baby is the most beautiful baby in the world (IB) | Mitt barn är det vackraste barnet i världen | My child is the most beautiful child in the world |
| I wish my baby would somehow go away (IB) | Jag önskar att mitt barn på något sätt skulle försvinna | I wish that my child would somehow disappear |
| My baby makes me feel anxious (AC) | Mitt barn gör mig nervös | My child makes me feel nervous *(anxious, uneasy)* |
| I am afraid of my baby (AC) | Jag är rädd för mitt barn | I am afraid of my child |
| My baby annoys me (RA) | Mitt barn stör mig | My child annoys *(bothers)* me |
| I feel confident when caring for my baby (AC) | Jag känner mig säker när jag tar hand om mitt barn | I feel confident *(secure)* when I take care of my child |
| I feel the only solution is for someone else to look after my baby (RA) | Jag känner att den enda lösningen är att någon annan ser efter mitt barn | I feel that the only solution is for someone else to take care of my child |
| My baby is easily comforted (AC) | Mitt barn är lätt att trösta | My child is easy to cheer up *(comfort)* |

**SI 2** The translation of the Postpartum Bonding Questionnaire (PBQ) (Brockington et al., 2001) into Swedish and the back-translation to English.

IB=impaired bonding subscale (12 items), RA=rejection and anger subscale (7 items), AC=anxiety about care subscale (4 items). The risk of abuse

subscale (2 items) was not included.
